# Considerations for partnering with Ryan White Case Managers to create equitable opportunities for people with HIV to participate in research

**DOI:** 10.1371/journal.pone.0276057

**Published:** 2022-10-19

**Authors:** Elizabeth Lockhart, DeAnne Turner, Jerome T. Galea, Stephanie L. Marhefka

**Affiliations:** 1 Center for Health Policy and Health Services Research, Henry Ford Health System, Detroit, Michigan, United States of America; 2 School of Nursing, University of South Florida, Tampa, Florida, United States of America; 3 School of Social Work, University of South Florida, Tampa, Florida, United States of America; 4 College of Public Health, University of South Florida, Tampa, Florida, United States of America; 5 Department of Global Health and Social Medicine, Harvard Medical School, Boston, Massachusetts, United States of America; University of Minnesota Twin Cities: University of Minnesota Twin Cities, UNITED STATES

## Abstract

Many research studies focus on recruitment from one or few HIV clinics or internet-engaged populations, but this may result in inequitable representation of people with HIV (PWH), across the rural/urban/suburban continuum. Ryan White Case Managers (RWCM) meet regularly with PWH, potentially positioning them as partners in gathering research-related data from diverse groups of low-income, marginalized, PWH. Yet, data collection in partnership with RWCM, particularly over large geographic areas, has been under-explored. We partnered with RWCM and their organizations throughout Florida to administer a 10-item technology use and willingness survey to clients living with HIV; RWCMs provided process-oriented feedback. Among 382 approached RWCM, 71% completed human subjects and survey administration training; 48% gathered data on 10 predetermined survey administration days; and 68% administered at least one survey during the entire period for survey administration. Altogether, 1,268 client surveys were completed, 2.7% by rural participants. Stigma, privacy concerns, and disinterest reportedly inhibited *client* participation; competing obligations, policies, and narrow recruitment windows prevented some RWCM from offering the survey to clients. Research should further explore strategies and best practices to ensure equitable access to participate in research among PWH.

## Introduction

Social and behavioral research is critical to understand the needs of people with HIV (PWH) as well as potential ways to effectively address those needs. After more than 30 years of HIV-related research, ensuring representation of different groups of PWH in social and behavioral research is still an issue. Historically, those who are rural-residing and minority populations tend to be underrepresented in research [1, 2]. When people are under-represented in research, it can lead to findings that are not generalizable to certain sub-populations and can create problems related to distributive justice, that is, the fair, equitable, and appropriate distribution of healthcare resources.

Current recruitment methods in HIV-related research have limitations. Traditional venues/media for recruiting PWH to participate in research include clinics and/or community-based sites, or print media (e.g. flyers), and the internet. Internet-based recruitment can reach large numbers of PWH, but tends to underrepresent people of color [3, 4]. Additionally, men–particularly gay, bisexual, and other men who have sex with men [5, 6]–are more likely to be recruited via the internet compared to women [5, 6]. Internet-based data collection may also under-sample people without reliable internet access [7, 8] or adequate computer skills [7, 8].

Recruitment at clinics and community-based organizations may lead to more diverse samples than those recruited online [3]. However, recruitment at these sites is often costlier and less effective at reaching large numbers of PWH than internet-based recruitment [9, 10]. We sought a recruitment strategy to leverage aspects of both approaches—the high numbers of participants from internet-based recruitment and demographic diversity afforded by clinic-based recruitment—by working with direct service personnel within the Ryan White Care Program, one of the U.S.’s biggest providers of HIV care services. Serving over 500,000 PWH in 2018, Ryan White Case Managers (RWCM) reach over half of all PWH in the U.S. [11]. As of 2020, over 110,000 PWH lived in Florida, with 53,000 receiving Ryan White services—making Florida an ideal location to reach large numbers of PWH [12].This approach may increase opportunities for participation from people not well connected to academic medical centers, living in rural areas, and who may have limited technological access.

Prioritizing distributive justice so that all people have the opportunity to participate in research is critical and necessary. RWCM could play a critical role in ensuring distributive justice for PWH. As gatekeepers to large numbers of PWH from diverse backgrounds, RWCM could be trained to recruit PWH and collect research-related data. Increasing the representation of all PWH in research, and not just those who typically participate, may help increase programming, and the allocation of resources to all. Ryan White Care Program recipients comprise uninsured and underinsured persons, most of whom earn <100% of the federal poverty level and live throughout all regions of the United States [11]. RWCM provide medical and social services referral and coordination to clients who must renew their eligibility every six months to one year. RWCM are in regular contact with a large, racially and geographically diverse cohort of PWH, including those traditionally underrepresented in HIV research, such as people who are socioeconomically disadvantaged and/or disenfranchised [13].

The feasibility and acceptability of RWCM-conducted participant recruitment, screening, and data collection for research-related purposes is unknown. Previous work seeking to understand RWCM’s role in research has included RWCM participation in programmatic needs assessments [14, 15] and referral of participants for research studies [16]. However, research has not explored RWCM’s uptake of research involvement, or related feasibility and acceptability. On the one hand, because RWCM interact with their clients regularly and, as a result, may develop strong rapport with clients [17], they are uniquely positioned to recruit PWH for research; however, RWCM have multiple demands and may have limited time to help their clients participate in research. Additionally, there is the potential that participants may feel coerced to participate in research if requested to do so by their RWCM. Steps are needed to make sure that clients feel empowered to make an informed decision about participating in research.

The primary purpose of this paper is to determine the facilitators and barriers to successful engagement of RWCM in recruitment and data collection. Additionally, we sought to 1) identify the rate of voluntary participation of RWCM to assist with recruitment and data collection in a statewide study of technology use among PWH; 2) determine if RWCM in Florida achieve a survey completion rate equivalent to five surveys per known statewide case manager on five pre-determined days; and 3) test if RWCM data collection results in a rural patient participant rate proportionate to the percent of rural PWH estimated to be living in Florida (+/- 5%).

## Materials & methods

### RWCM recruitment & training

A letter of support was requested from the HIV/AIDS Section Administrator at the state department of health and sent to all Ryan White Part A (funding designated for counties/cities most affected by HIV) and Ryan White Part B (funding designated to states and territories to provide HIV health care and support services) Lead Agencies (agencies that receive Ryan White Parts A and B funding directly from the state). Subsequently, the principal investigator contacted each Ryan White Lead Agency, seeking verbal consent to assist with the project and asking for a list of their Ryan White case management contracted agencies. We contacted executive directors and/or case manager supervisors to request their agreement to connect our staff with their RWCM teams. While the overarching study received IRB approval from the Florida Department of Health, this sub-study was considered to be quality improvement data collection and therefore not considered research.

At each case management agency, our primary contact gave the RWCM information about the study and the study website [which included information about the study, required training modules (human subjects and survey administration), and the client survey]. RWCM completed a no-cost human subjects online training module, which covered codes and regulations, respect for persons, beneficence, and justice. Consistent with IRB approval and to prevent coercion: 1) RWCM were not incentivized based on the number of clients who agreed to participate; 2) RWCM obtained informed consent from each participant; and 3) the voluntary nature of the study was underscored. We offered trainings online and in-person when geographically feasible. Following each training, RWCM completed an assessment to demonstrate proficiency, which then generated a certificate of completion. Once fully trained, RWCM could administer the client survey.

We offered each Lead Agency $1000 to support data collection. Lead Agencies were incentivized, rather than contracted case management agencies, to prevent coercion from the organizational level to RWCM. Each Lead Agency could then decide how to utilize the incentive. We came to this decision after considering state/federal regulations on payment, and consulting with local case management supervisors and one Lead Agency on the best means of incentivizing RWCM participation. Based on case management supervisor input, lead agencies were encouraged to make those funds available to RWCM teams to cover client needs that could not be otherwise met. No agency employees received direct compensation for involvement. We offered each agency access to the aggregate data from their organization, region, and state so it could be used for grant applications, reports, or other purposes.

### Primary data collection

This cross-sectional study used 3 rounds of data collection.

#### Round 1

During April-May, 2016, five days were designated in which RWCM were requested to invite all clients with whom they had contact that day to complete a 10-question survey regarding client technology use and willingness. Five questions were demographic questions and five questions were adapted from the U.S. Census American Community Survey [18]. Five predetermined survey days were selected in an attempt to reduce additional burden on the RWCM. Trained RWCM received email reminders to engage clients in surveys on those days.

#### Round 2

Because the target number of client surveys was not met after Round 1 (see results: *Round 1 Survey Completion Rate and Rural Participation Rate)* we added an additional five recruitment days June-July 2016; email announcements were made. Clients who completed the survey in both Rounds 1 and 2 could indicate desire to be entered into a raffle to win a $15 gift card.

#### Round 3

After Rounds 1 and 2, and based on RWCM feedback, the survey was made available to all trained RWCM on all days and times from July 2016 to April 2017. The survey was re-opened to allow RWCM the time to offer the survey to all of their clients and to reach recruitment goals. Monthly emails were sent to RWCM and case management supervisors reminding them about the survey and regional progress towards study recruitment goals. Additionally, research staff contacted organizations in areas where the sample size goal was not met and offered to complete surveys with clients at pre-existing on-site events (e.g. holiday party, group meetings).

### Secondary data collection

To understand experiences administering the survey, an anonymous 9-question, online questionnaire was emailed to RWCM the day after each survey administration date in Round 1. Two open-ended questions were included: 1) *If you did not administer the survey at all or had some clients not complete the survey*, *what were the reasons*? and *2) If clients said they did not want to take the survey*, *what were the reasons they told you for not wanting to take the survey*? Further, RWCM reported the total number of Ryan White clients in their caseload; method used to record client responses (computer, mobile device, paper); number of clients with whom they interacted on the survey day; and number of surveys administered on the survey day. Following questionnaire completion, RWCM could opt into a raffle for a $15 gift card.

### Analysis

This analysis focused on the participation of RWCM in the research process. To see the main results of the participant surveys, please see [19, 20]. Quantitative data frequencies were generated in SPSS v.24 [21]. Rurality was calculated based on a participant’s self-reported ZIP code and data obtained from the Department of Agriculture Rural-Urban Commuting Area (RUCA). ZIP codes were matched to the Federal Information Processing Standards code crosswalk file, which were then matched to a RUCA code (1 = urban, 2–6 = suburban, 7–10 = rural). The number of PWH in each state ZIP code was obtained from the state department of health. The percentage of PWH living in rural areas of the state was compared to the percentage of PWH living in rural areas who completed the survey. Text-based responses outlining the RWCM experiences were analyzed using MAXQDA [22] by two reviewers [E.L and D.T.]. First, reviewers read the responses and generated a codebook. Each reviewer coded 10% of responses and both reviewers revised the codebook in an iterative process until agreement was attained. The primary author [E.L.] coded the remaining responses. Summaries of the thematic analysis [23] were developed based on organization/location and number of surveys completed.

## Results

In total, across the entire study time period, 1,268 surveys were completed.

### Round 1 RWCM participation rate

**Fig 1:**
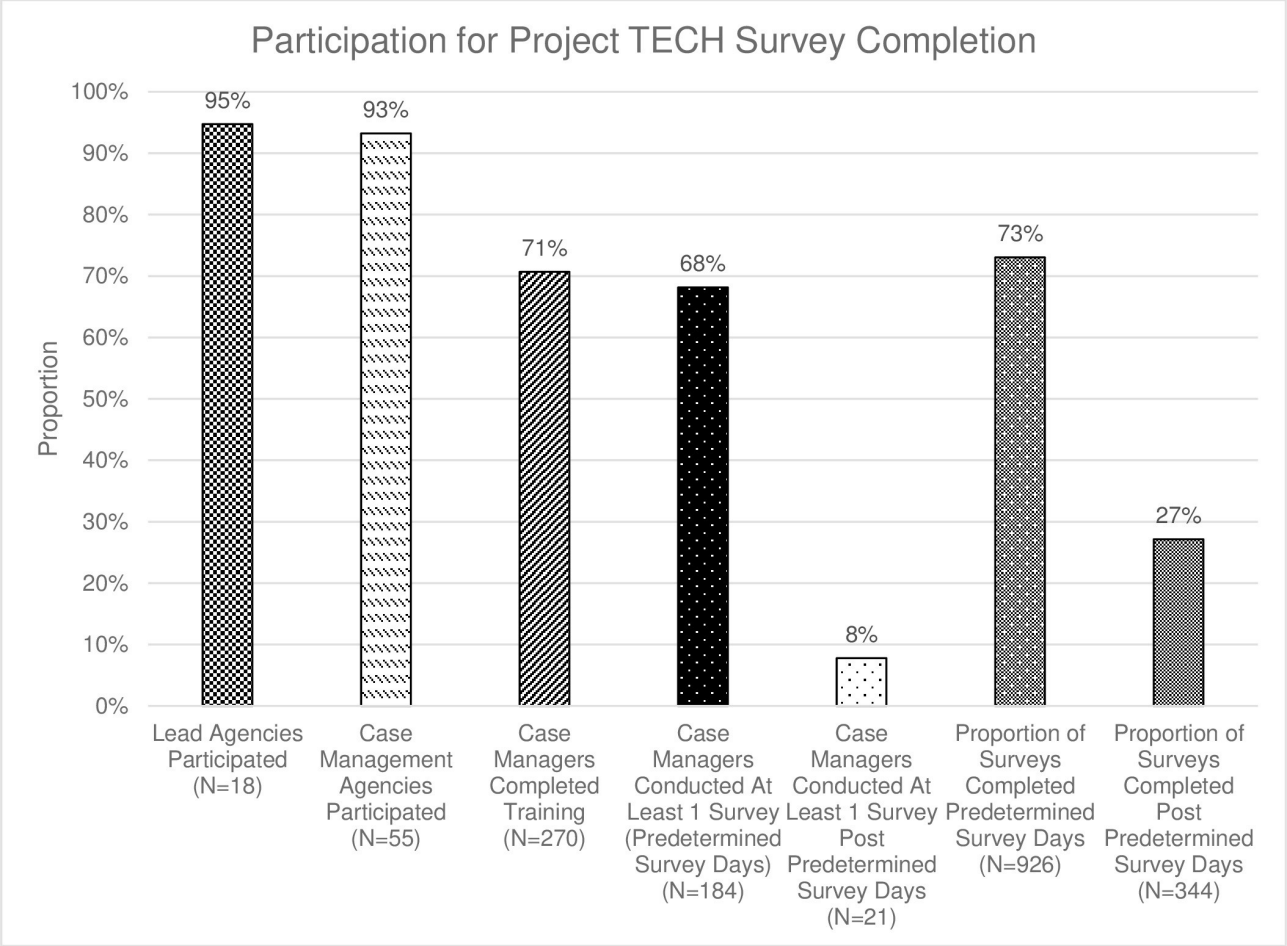
Case manager participation flowchart.

Eighteen of the 19 Ryan White Lead Agencies in the state participated by sharing information with their contracted case management agencies or having internal RWCM offer the survey to clients (Fig 1). Overall, 55 of 59 contracted agencies provided names and contact information for 382 RWCMs (Table 1). Emails were sent to all 382 RWCMs containing information about participation and training. Two hundred and seventy (70.7%) RWCMs from organizations throughout the state completed the human subjects and survey administration trainings. These RWCMs represented 48 of the 55 organizations, serving 47 of 67 state counties.

**Table 1:** Ryan White Case Managers (RWCM) who administered surveys.

	Number of RWCM Who Administered Surveys		Number of RWCM Who Completed Process Surveys	
	n	%	n	%
1 Day	74	40.2%	46	29.7%
2 Days	50	27.2%	37	23.9%
3 Days	26	14.1%	26	16.8%
4 Days	15	8.2%	11	7.1%
5 Days	10	5.4%	17	11.0%
6 Days	6	31.6%	9	5.8%
7 Days	2	10.5%	3	1.9%
8 Days	2	10.5%	5	3.2%
Ever, Post Day 5	10	52.6%	18	11.6%
Ever, Throughout the Study	184	68.1%^c^	155	84.2%^d^

^a^ Days 1–5 denominators are 184, the number of RWCM who completed at least one client survey

^b^ Days 6–8 denominators are 19, the number of RWCM who completed at least one client survey post predetermined survey dates

^c^ Denominator is 270, the number of CMs who completed trainings

^d^ Denominator is 184, the number of CMs who completed at least one client survey

### Round 1 survey completion rate and rural participation rate

In Round 1, RWCM (n = 184) completed 755 surveys across the five predetermined survey dates. RWCM completed an average of five surveys across the predetermined days (SD = 4.40; range = 1–34). Of the known case managers in the state (n = 382), an average survey completion rate of 1.98 surveys per RWCM was achieved. RWCM completed surveys with 40 PWH (2.7%) who lived in rural areas, more than the proportion of PWH living in rural areas (1.3%) in the state [24]. RWCM completed surveys with 171 (22.6%) clients who were White, 210 (27.8%) clients who were Hispanic, 316 (41.9%) clients who were Black.

155 RWCM (57.4% of trained RWCM) completed the process survey in which they reported meeting with 0–55 clients per survey day (Median = 3) and administering the survey to 0–15 clients per reporting day (Median = 1). Of those, most RWCM (70.7%) completed between 1 and 5 surveys with their clients per reporting day; 73% entered responses via a computer, 12% on paper, and 1% via a mobile device.

#### Reasons for survey non-completion

Lack of RWCM time was a salient reason RWCM did not complete the survey. For example: *I don’t know if other Ryan White case managers also do eligibility—in [our] County*, *we are both eligibility workers and case managers—we struggle to do both jobs so are extremely busy*.

Some RWCMs reported they did not interact with clients on the survey day, due to competing administrative duties (e.g., paperwork, meetings) or client no-shows. One RWCM said, “*I did not come into contact with any clients…I spent my day working on projects for HOPWA [Housing Opportunities for People with AIDS]*.” Some RWCMs said clients “*did not show up for appointments*”.

RWCMs reported clients did not complete the survey because they “*just refused*” and did not provide information as to why. At times, clients explicitly told RWCMs they were not interested. Some clients “*declined to do the survey via phone due to loss of minutes*”; that is, they were unable or unwilling to use their cellular plan time for the survey: *“[Clients] did not have sufficient minutes left on their phones*.” Other clients refused because they were in transit or on a work break while speaking to their RWCM. One RWCM reported, “*…they were at work and could only speak for a minute*.” Although RWCMs were asked to offer the survey to each client encountered, sometimes RWCMs did not offer the survey when they knew their client was at work.

Stigma, privacy concerns, and not wanting to inadvertently disclose their HIV status were reasons clients refused the survey. One RWCM reported, “*Some clients did not want to participate due to [the] fear of disclosure issue [of HIV status to co-workers]*.” Another shared:

*Many clients do not wish to participate in the survey because of various socio-economic and socio-cultural reasons*. *Most simply do not wish to interact with ‘HIV care’ more than they have to…Many simply do not wish to engage beyond the minimum requirement due to their own personal reasons*.

#### RWCM feedback regarding the process

Some RWCM reported the 10-item client survey was too long. One wrote, “*May be a bit too long*, *comments [were] made by a few clients when completing today*.” Although our training stressed that the survey should be offered to all clients, other RWCM noted the survey was possibly inappropriate for their clients because they were “*not computer literate*,” a finding especially prevalent in organizations serving rural clients. One RWCM shared, “*The one client I did complete the survey with is computer and internet illiterate so all the questions after question X [technology-based questions] up to Y [demographic questions] [are] irrelevant*.*”*

Several RWCM reported the survey provided them an additional tool for understanding clients. One wrote, “*The survey is a good idea*. *It opens a door for us to get to know our clients more*.” Specifically, it helped them learn about client technology access. “*I enjoyed administering the survey*. *Most clients used internet more than I expected and some not at all*.”

### Round 2 RWCM participation rate

In Round 2, we designated an additional five dates in which RWCMs could complete the survey with their clients. During those five days, 50 RWCMs (8 new and 42 previously engaged RWCMs) completed surveys with their clients.

### Round 2 survey completion rate and rural participation rate

169 surveys were completed during Round 2. An average of 3.34 surveys were completed (SD = 3.47); this is n = 2.04 per trained RWCM (including those who never completed a survey across the entire study) and n = 0.92 per trained RWCM who completed at least one survey during the study. Surveys were completed with 4 (0.3%) rural-residing PWH. Completed surveys were taken by 31 (18.3%) White clients, 48 (28.4%) Hispanic clients, and 83 (49.1%) Black clients.

### Round 3 RWCM participation rate

RWCM made clear they preferred no restrictions on when they could administer the survey to clients. Thus, when we moved to Round 3 and asked RWCM in geographic areas that had not met recruitment goals to continue to offer the survey to their clients, we made no restrictions on when it could be administered. Geographic areas that had met their recruitment goals were not included in Round 3. Once survey days were unrestricted (Round 3), 20 RWCM (11 new and 9 previously engaged RWCM) administered surveys to their clients. One organization designated a RWCM to complete surveys with all clients accessing services at their organization; this RWCM completed 104 surveys with clients over three months. Additionally, our research staff collected 96 surveys with clients at three organizations during large, client-centered on-site meetings.

### Round 3 survey completion rate and rural participation rate

**Fig 2:**
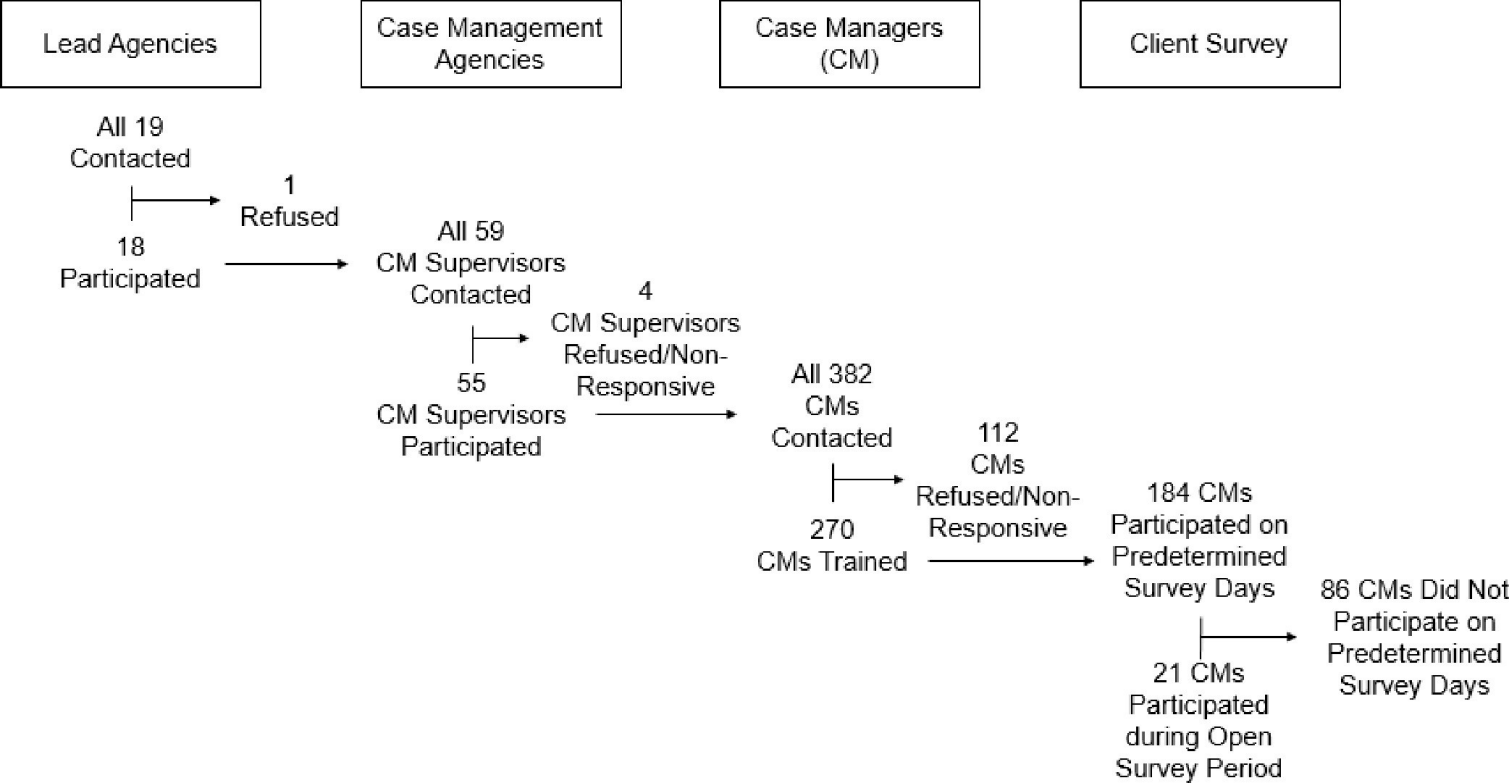
Proportion of participation for survey completion. Note: Not all CMs were invited to participate in Post predetermined survey days due to completion of survey goals.

During Round 3, 344 surveys were completed. Excluding the surveys completed by the one RWCM designated to administer surveys and our study staff, per participating RWCM an average of 7 surveys were completed during Round 3 (SD = 9.96); this is n = 1.27 per trained RWCM (including those who never completed a survey across the entire study) and n = 1.87 per trained RWCM who completed at least one survey during the study (Table 1) (Fig 2). RWCM completed surveys with 2 PWH (0.1%) who lived in rural areas. Surveys were completed with 36 (10.5%) White clients, 58 (16.9%) Hispanic clients, and 231 (67.2%) Black clients.

## Discussion

Findings of the present study suggest partnering with RWCM may be acceptable and feasible for research data collection and may also benefit RWCM and the organizations in which they work. Success of the approach may vary based on flexibility and expectations of both participants and the RWCM and organizations where they work. Involving RWCM allowed us to obtain a large, diverse sample of PWH. RWCM from 93% of all state RWCM agencies conducted a least one survey with their client(s), with a total of 1,268 surveys completed. Yet lack of time, competing administrative duties, and not seeing clients on the pre-determined survey days led to fewer surveys completed. Some clients refused the survey, while others were too busy. Some clients were concerned about stigma and privacy. However, partnering with RWCM was successful in collecting research data among PWH.

Partnering with RWCM is an effective approach for data collection among PWH and has the potential to provide benefits to all involved. The benefits of RWCM connecting researchers to PWH from geographically diverse areas are many, as patient care may be improved based on outcomes of the research and researchers are able to understand the experiences of a geographically representative sample. Engaging RWCM in data collection may give a voice to PWH who may otherwise not have the opportunity to share their experiences. Moreover, having RWCM help with research, similar to Community Research Navigators (community health leaders engaged to widen the research participation of ethnic minority populations in health-related research) [25], may increase distributive justice. More PWH would have the opportunity to participate in research and have their voices heard. Hard to reach, disenfranchised, and underrepresented populations may benefit most from RWCM involvement in these opportunities. Finally, when remuneration is provided, it can be a source of assistance to the Ryan White clients with few resources.

Identifying the most efficient means of conducting RWCM ethics and study administration training was complex because many organizations preferred in-person trainings. However, we could not travel to all agencies due to distance, time, and limited funding. Completion of the online training was likely biased towards the most motivated RWCM. Because training was required before RWCM could administer client surveys, some clients (assigned to RWCM who did not complete our training) were left without opportunity to participate. Finally, RWCM were not allowed to participate in research-related data collection during their Ryan White Program funded time, which likely limited survey administration. Understanding potential changes in funding streams to cover RWCM research-related activity would be a useful future research direction.

Each group of people affected by HIV has different experiences and outcomes that may be missed if they are not included in research (e.g., men who have sex with men, African Americans and Latinos of all sexual orientations, transgender individuals, and those living in rural areas). RWCM may be able to integrate research opportunities as opportunities arise in their routine interactions with their clients. As a result, RWCM may get to know their clients better and help their clients access additional resources (e.g. gift cards, bus passes) for their daily lives. However, to the best of our knowledge, RWCM have not been included in participant screening, informed consent, and data collection for research-related purposes. The aim of this study was to investigate the process and barriers to collaborating with RWCM in collecting research-related data, which may ultimately lead to increased participation in research opportunities. Previous research has found that recruiting PWH via technologies leads to unequal representation and attrition, specifically by underrepresentation of people of color and women [5, 6, 26, 27]. Partnering with RWCM may increase access to populations underrepresented in research due to the reach of Ryan White programming and the rapport RWCM have with their clients. Future research should examine the perceptions of Ryan White clients in participating in research with their RWCM.

The benefits to partnering with RWCM outweighed the drawbacks. The typical RWCM job description does not include research-related data collection, so this was an added duty. While we tried to make sure the benefits for both the RWCM and their clients were appropriate (e.g., each client had the chance for a gift card via a raffle; RWCM would learn more about their clients), the typical caseload and duties of RWCM may have precluded them from participating. Concerns regarding multiple job tasks and limited time have been noted as a barrier to participate in research by other non-traditional research staff, including community health workers [28, 29]. Further, RWCM are not currently funded to collect research-related data, this may require a change in payment systems if data collection were regularly included in a job description; for example, grant-funding has supported community health workers participating in data collection [30]. Future research should examine how to incorporate research-related data collection from the perspectives of organizational leadership at the agencies providing RWCM. Despite the drawbacks, partnership with RWCM is an effective way to reach large numbers of PWH.

Ethics training also presented challenges. The completion of human subjects training by RWCM was required by the IRB. Although hiring guidelines may vary, RWCM should be well-versed in confidentiality requirements and protections [31]. Asking RWCM to complete ethics training related to privacy and confidentiality of their clients may be unnecessary since they are already required to maintain strict client privacy and confidentiality as part of their job. However, other aspects of ethics may be particularly important to emphasize when training non-traditional research staff like RWCM. Autonomy must be emphasized to ensure non-coercion. The necessity of emphasizing the voluntary nature of the study was reiterated within our trainings, consent form, and the study website. Importantly, justice should be emphasized in ethics training with RWCM so clients are not differentially invited to participate in research. Given the unique position of RWCM and other community-based health-related workers, alternative ethics training models may be beneficial [32, 33]. Customizing ethics training to this population could ease the training burden on RWCM while still providing the knowledge and skills necessary to conduct research.

Limitations of our study include that our study was restricted to one state. Additionally, RWCM who did not participate in the process survey may have had reasons for non-participation different than those reported here. Lack of client refusal data from RWCM who did not participate in the process survey posed challenges for reporting and determining generalizability. The client survey was administered about 5 years ago and prior to COVID-19; engagement with technology and HIV services may now be different. Similarly, because the client survey focused on technology use, participation rates may have been higher than they would have been with a more stigmatized topic (e.g., substance use). Moreover, some RWCM reported not inviting all clients to take the survey, citing time constraints. Some incorrectly believed some clients were ineligible because they did not use technology—such misperceptions can skew data.

For researchers, partnering with RWCM for data collection is feasible, acceptable, and mutually beneficial. Studies are needed to test whether RWCM data collection is more cost-effective than clinic-based recruitment for gathering sensitive information and ensuring larger sample sizes. Moreover, the literature will benefit from additional studies that test various implementation strategies for RWCM engagement in recruitment and data collection.

## Supporting information

S1 Appendix (DOCX)
