# Supplementary material for: Considerations for partnering with Ryan White Case Managers to create equitable opportunities for people with HIV to participate in research
Source: PLoS One. 2022 Oct 19;17(10):e0276057. doi: 10.1371/journal.pone.0276057 (PMC9581377; doi:10.1371/journal.pone.0276057)
Supplement: S1 Appendix — (DOCX) [file pone.0276057.s001.docx]

**Appendix**

1. **Project TECH 10-Question Survey**
2. What is your ZIP code?
3. How old are you in years?
4. The next question is about your race and ethnicity. Tell me yes or no for each one. Are you:
   1. Hispanic or Latino?
   2. Black or African American?
   3. White or Caucasian?
   4. Asian or Other Pacific Islander?
   5. Native American?
5. What is your gender? Please say yes or no for each one. Are you:
   1. Female?
   2. Male?
   3. Trans Male?
   4. Trans Female?
   5. Another gender?
6. Now I have a question about your sexual experience. Tell me yes or nor for each one.
   1. Have you ever had sex with a man?
   2. Have you ever had sex with a woman?
7. Now I am going to ask about technology. Please tell me yes or no for each one. Do you use:
   1. A desktop, laptop, netbook, or notebook computer?
   2. A tablet computer like an iPad, Samsung Galaxy, or Windows Tablet?
   3. A mobile phone or cellphone with text messaging?
   4. Apps on your mobile phone or cellphone?
   5. The internet on your mobile phone or cellphone?
   6. Another type of computer (Write in)
8. Do you ever use the internet or email:
   1. At home?
   2. Outside of your home?
9. Do you or any member of you household access the internet at home using the following types of connections?
   1. A subscription to an internet service?
   2. A mobile phone or cellphone?
   3. Dial up service?
   4. Internet service like DSL, cable modem, fiber-optic, or satellite internet?
10. Do you ever use the internet to do any of the following activities?
    1. Send or receive email?
    2. Do an online search?
    3. Use social networking sites like Facebook, Myspace, Instagram, or Twitter?
    4. Video-chat like with Skype, Oovo, Facetime, or another program?
    5. Get information about HIV?
    6. Communicate with other people who are living with HIV?
    7. Find partners or companions online through sites like POZ Personals, Match.com, Grindr, OkCupid, OurTime, or Tinder?
11. Earlier I asked you about the devices you have used in the past, now I will ask you about devices you would ever be willing to use. Would you EVER be willing to use the following devices to access information about HIV?
    1. A desktop, laptop, netbook, or notebook computer?
    2. A tablet computer like an iPad, Samsung Galaxy, or Windows Tablet?
    3. A mobile phone or cellphone with text messaging?
    4. Apps on your mobile phone/cellphone?
    5. The internet on your mobile phone/cellphone?
    6. Another type of device or computer (write in)?
12. **RWCM Process Survey Questions**
13. How many Ryan White Case management clients do you currently have in your caseload?
14. Are you funded by the following (select all that apply):
    1. Ryan White Part A
    2. Ryan White Part B
    3. Ryan White – Other
15. How did you administer the survey?
16. How many clients did you meet with via phone or in person on [Date of Survey]?
17. How many clients who you met with via phone or in person on [Date of Survey] were specifically Ryan White clients?
18. How many Ryan White Case Management clients did you administer the survey to on [Date of Survey]?
19. How many Ryan White Case Management clients did you NOT administer the survey to on [Date of Survey]?
20. If you did not administer the survey at all or you had some clients not complete the survey, what were the reasons?
21. If clients said they did not want to take the survey, what were the reasons they told you for not wanting to take the survey?
